# Development of COVIDVax Model to Estimate the Risk of SARS-CoV-2–Related Death Among 7.6 Million US Veterans for Use in Vaccination Prioritization

**DOI:** 10.1001/jamanetworkopen.2021.4347

**Published:** 2021-04-06

**Authors:** George N. Ioannou, Pamela Green, Vincent S. Fan, Jason A. Dominitz, Ann M. O’Hare, Lisa I. Backus, Emily Locke, McKenna C. Eastment, Thomas F. Osborne, Nikolas G. Ioannou, Kristin Berry

**Affiliations:** 1 Division of Gastroenterology, Veterans Affairs Puget Sound Healthcare System, University of Washington, Seattle; 2 Research and Development, Veterans Affairs Puget Sound Health Care System, Seattle, Washington; 3 Division of Pulmonary, Critical Care, and Sleep, Veterans Affairs Puget Sound Healthcare System, University of Washington, Seattle; 4 Division of Nephrology, Veterans Affairs Puget Sound Healthcare System, University of Washington, Seattle; 5 Department of Veterans Affairs, Population Health Services, Palo Alto Healthcare System, Palo Alto, California; 6 Division of Allergy and Infectious Diseases, Veterans Affairs Puget Sound Healthcare System, University of Washington, Seattle; 7 Veterans Affairs Palo Alto Healthcare System, Palo Alto, California; 8 Department of Radiology, Stanford University School of Medicine, Stanford, California; 9 Paul G. Allen School of Computer Science and Engineering, University of Washington, Seattle

## Abstract

**Importance:**

A strategy that prioritizes individuals for SARS-CoV-2 vaccination according to their risk of SARS-CoV-2–related mortality would help minimize deaths during vaccine rollout.

**Objective:**

To develop a model that estimates the risk of SARS-CoV-2–related mortality among all enrollees of the US Department of Veterans Affairs (VA) health care system.

**Design, Setting, and Participants:**

This prognostic study used data from 7 635 064 individuals enrolled in the VA health care system as of May 21, 2020, to develop and internally validate a logistic regression model (COVIDVax) that predicted SARS-CoV-2–related death (n = 2422) during the observation period (May 21 to November 2, 2020) using baseline characteristics known to be associated with SARS-CoV-2–related mortality, extracted from the VA electronic health records (EHRs). The cohort was split into a training period (May 21 to September 30) and testing period (October 1 to November 2).

**Main Outcomes and Measures:**

SARS-CoV-2–related death, defined as death within 30 days of testing positive for SARS-CoV-2. VA EHR data streams were imported on a data integration platform to demonstrate that the model could be executed in real-time to produce dashboards with risk scores for all current VA enrollees.

**Results:**

Of 7 635 064 individuals, the mean (SD) age was 66.2 (13.8) years, and most were men (7 051 912 [92.4%]) and White individuals (4 887 338 [64.0%]), with 1 116 435 (14.6%) Black individuals and 399 634 (5.2%) Hispanic individuals. From a starting pool of 16 potential predictors, 10 were included in the final COVIDVax model, as follows: sex, age, race, ethnicity, body mass index, Charlson Comorbidity Index, diabetes, chronic kidney disease, congestive heart failure, and Care Assessment Need score. The model exhibited excellent discrimination with area under the receiver operating characteristic curve (AUROC) of 85.3% (95% CI, 84.6%-86.1%), superior to the AUROC of using age alone to stratify risk (72.6%; 95% CI, 71.6%-73.6%). Assuming vaccination is 90% effective at preventing SARS-CoV-2–related death, using this model to prioritize vaccination was estimated to prevent 63.5% of deaths that would occur by the time 50% of VA enrollees are vaccinated, significantly higher than the estimate for prioritizing vaccination based on age (45.6%) or the US Centers for Disease Control and Prevention phases of vaccine allocation (41.1%).

**Conclusions and Relevance:**

In this prognostic study of all VA enrollees, prioritizing vaccination based on the COVIDVax model was estimated to prevent a large proportion of deaths expected to occur during vaccine rollout before sufficient herd immunity is achieved.

## Key Points

**Question:**

How can the risk of SARS-CoV-2–related death be estimated in the general population to be used for vaccination prioritization?

**Findings:**

In this prognostic study of more than 7.6 million individuals enrolled in the Veterans Affairs health care system, a logistic regression model (COVIDVax) was developed to estimate risk of SARS-CoV-2–related death using the following 10 characteristics: sex, age, race, ethnicity, body mass index, Charlson Comorbidity Index, diabetes, chronic kidney disease, congestive heart failure, and the Care Assessment Need score. The model was estimated to save more lives than prioritizing vaccination based on age or on the US Centers for Disease Control and Prevention vaccination allocation.

**Meaning:**

These findings suggest that prioritizing vaccination based on the model developed in this study could prevent a substantial number of SARS-CoV-2–related deaths during vaccine rollout.

## Teaser

This prognostic study develops a model that estimates the risk of SARS-CoV-2–related mortality among all enrollees of the US Department of Veterans Affairs (VA) health care system.

## Introduction

Highly efficacious vaccines against SARS-CoV-2 have received emergency use authorization from the US Food and Drug Administration (FDA).^1,2,3^ Vaccine supply is expected to be limited initially. Logistical challenges (eg, cold storage and 2-dose requirements) may further prolong the time needed to vaccinate most of the US population. The US Centers for Disease Control and Prevention (CDC) Advisory Committee on Immunization Practices (ACIP) outlined ethical principles that should guide allocation given limited supply and recommended a phased approach to vaccine allocation: phase 1a, health care personnel and long-term care facility (LTCF) residents; phase 1b, frontline essential workers and persons aged 75 years or older; phase 1c, essential workers, persons aged 65 to 74 years, and persons aged 16 to 64 years with high-risk medical conditions; and phase 2, which includes the remaining population.^4,5,6^

Prioritizing persons for vaccination according to their risk of SARS-CoV-2–related death would minimize the number of SARS-CoV-2–related deaths that would occur in the time it takes to vaccinate a large enough proportion of the US population to achieve sufficient herd immunity.^7^ We aimed to develop a model that estimates the risk of SARS-CoV-2–related death in the general population (the COVIDVax model) and to estimate the number of SARS-CoV-2–related deaths prevented by prioritizing vaccination based on our model vs an approach based on age alone (ie, oldest first) or based on the ACIP-recommended phases of vaccination.

## Methods

### Study Population and Data Source

We identified all persons aged 18 years or older who were alive and enrolled in the Veterans Affairs (VA) health care system as of May 21, 2020 (n = 7 655 212). We excluded data from the early months of the pandemic to increase the relevance of our model to contemporary practice. We excluded 6596 veterans who had tested positive for SARS-CoV-2 more than 30 days before May 21, 2020 (ie, before April 21, 2020). We also excluded 13 552 persons who were residents in VA LTCFs or nursing homes during the study period, because these individuals would already be getting vaccinated in phase 1a, resulting in a study cohort of 7 635 064 persons.

We used data from the VA Corporate Data Warehouse (CDW), a relational database of VA enrollees’ electronic health records (EHRs), developed by the VA Informatics and Computing Infrastructure (VINCI) to support research and clinical operations. The study was approved by the VA Puget Sound institutional review board, which granted a waiver of informed consent because this was a database-derived study. This study followed the Strengthening the Reporting of Observational Studies in Epidemiology (STROBE) reporting guideline.

### Definition of SARS-CoV-2–Related Death

Cohort members who tested positive for SARS-CoV-2 based on approved polymerase chain reaction tests and died of any cause within 30 days of their earliest positive test date were defined as having a SARS-CoV-2–related death.^8,9^ Deaths occurring both within and outside the VA are comprehensively captured in CDW through a variety of sources including VA inpatient files, VA Beneficiary Identification and Records Locator System (BIRLS), Social Security Administration (SSA) death files, and the Department of Defense.^10^

Cohort members were followed up for SARS-CoV-2–related death for 165 days (May 21 to November 2, 2020). Deaths occurring during this period were confirmed with updated death data through December 15, 2020, to allow additional time for deaths to be electronically recorded in CDW.

### Baseline Characteristics Considered for Inclusion in the COVIDVax Model

We only considered characteristics that are readily available in the EHR, including the following previously reported risk factors for adverse outcomes related to SARS-CoV-2^8,9,11,12,13^: age, sex, self-reported race and ethnicity, urban vs rural residence (based on zip codes), body mass index (BMI; calculated as weight in kilograms divided by height in meters squared), and Charlson Comorbidity Index (CCI), calculated using the Deyo^14^ modification of the CCI.^15^ We also considered the following 8 common preexisting medical conditions identified as high-risk conditions by the CDC,^16^ derived using diagnostic codes: chronic kidney disease (CKD), chronic obstructive pulmonary disease (COPD), cirrhosis, congestive heart failure (CHF), diabetes, hypertension, myocardial infarction (MI), and peripheral vascular disease (PVD). Finally, we considered the Care Assessment Need (CAN) score (version 2.5, 1-year mortality model), a validated measure of 1-year mortality in VA enrollees calculated using sociodemographic characteristics, clinical diagnoses, vital signs, medications, laboratory values, and health care utilization data from the VA national EHR.^17^ CAN scores range from 0 (lowest risk) to 99 (highest risk), corresponding to percentiles of risk among all VA enrollees. The CAN score was recently shown to be a predictor of COVID-19–related mortality.^18^

The value of each predictor was ascertained before the beginning of the observation period on May 21, 2020. We identified comorbid conditions recorded at any time before May 21, 2020. Values of the CAN score and BMI within 6 months before May 21, 2020 were used. For persons included in our analysis who tested positive for SARS-CoV-2 between April 21 and May 21, 2020, baseline characteristics were ascertained before the date of the earliest SARS-CoV-2–positive test.

Values were missing in source data for the CAN score (2 571 262 [33.7%]), urban/rural location (91 621 [1.2%]), and BMI (1 504 110 [19.7%]). Missing BMI values were deterministically imputed (eMethods in the Supplement). For urban/rural location and the CAN score, we used a missing category because we considered missingness to be potentially informative in an unbiased manner (eg, missing CAN score implies the VA enrollee was not assigned to a primary care team and was not hospitalized during the 6-month look-back period). We also developed a model that did not include the CAN score, which is only available in VA enrollees, so that our model could be subsequently validated in non-VA populations.

### Statistical Analysis

#### Model Development

Multivariable logistic regression was used to develop the COVIDVax model to estimate the risk of SARS-CoV-2–related death during follow-up using baseline patient characteristics. We used the following strategy to determine which of the 16 candidate predictor variables to include in our model. All variables with *P* < .05 in unadjusted models were evaluated for inclusion in the adjusted model and were dropped if the adjusted *P* > .05. Each dropped variable was reinserted sequentially during model construction and retained if it was significant; thus, we iteratively reassessed how each candidate predictor affected all others included in the model. We considered interactions between age and comorbidity but did not find they improved the model.

We contemplated using least absolute shrinkage and selection operator (LASSO) regression to perform variable selection and regularization. However, because these variables are listed by CDC-ACIP as high-risk criteria, we wanted to test each and explain why they were not included if they were dropped.

We also contemplated using machine learning modeling approaches. However, these are harder to execute in practice and might be perceived as lacking transparency (ie, the black box), which would reduce acceptability. All continuous variables (age, CCI, BMI, CAN score) were categorized as shown in Table 1. Analyses were conducted using Stata/MP version 16.1 (64-bit) statistical software (StataCorp).

**Table 1.** Associations Between Characteristics Included in the Prediction Model and SARS-CoV-2–Related Death in Training Subset Among 7 635 064 Veterans Affairs Enrollees

Characteristic	No. (%)		SARS-CoV-2 death rate during 5-mo period, per 10 000 individuals	Odds ratio		
	VA enrollees	SARS-CoV-2–related death		Crude	Adjusted COVIDVax model	
					With CAN score^a^	Without CAN score^b^
Sex						
Women	583 152 (7.6)	37 (1.9)	0.63	1 [Reference]	1 [Reference]	1 [Reference]
Men	7 051 912 (92.4)	1898 (98.1)	2.69	4.24 (3.06-5.87)	1.66 (1.19-2.32)	1.82 (1.31-2.53)
Age, y						
18 to <50	1 070 770 (14.0)	28 (1.4)	0.26	1 [Reference]	1 [Reference]	1 [Reference]
50 to <60	1 340 791 (17.6)	79 (4.1)	0.59	2.25 (1.46-3.47)	1.49 (0.96-2.32)	1.47 (0.96-2.27)
60 to <65	796 564 (10.4)	110 (5.7)	1.38	5.28 (3.49-8.00)	2.36 (1.52-3.66)	2.37 (1.56-3.61)
65 to <70	888 276 (11.6)	192 (9.9)	2.16	8.27 (5.56-12.29)	2.98 (1.94-4.60)	3.24 (2.16-4.84)
70 to <75	1 514 041 (19.8)	430 (22.2)	2.84	10.86 (7.41-15.92)	3.89 (2.56-5.93)	4.18 (2.83-6.18)
75 to <80	783 591 (10.3)	305 (15.8)	3.89	14.89 (10.11-21.93)	4.37 (2.84-6.72)	5.89 (3.97-8.76)
80 to <85	485 748 (6.4)	228 (11.8)	4.69	17.96 (12.13-26.59)	5.24 (3.39-8.11)	7.43 (4.97-11.11)
85 to <90	424 062 (5.6)	280 (14.5)	6.6	25.27 (17.13-37.26)	5.71 (3.69-8.85)	10.68 (7.16-15.93)
≥90	331 221 (4.3)	283 (14.6)	8.54	32.70 (22.18-48.22)	8.95 (5.78-13.87)	16.78 (11.24-25.04)
Race						
White	4 887 338 (64.0)	1288 (66.6)	2.64	1 [Reference]	1 [Reference]	1 [Reference]
Black	1 116 435 (14.6)	436 (22.5)	3.91	1.48 (1.33-1.65)	1.97 (1.76-2.21)	1.93 (1.73-2.16)
American Indian or Alaska Native	62 305 (0.8)	31 (1.6)	4.98	1.89 (1.32-2.70)	2.40 (1.68-3.43)	2.41 (1.69-3.45)
Other^c^	139 069 (1.8)	35 (1.8)	2.52	0.95 (0.68-1.34)	1.31 (0.93-1.83)	1.28 (0.91-1.79)
Declined, unknown, or missing	1 429 917 (18.7)	145 (7.5)	1.01	0.38 (0.32-0.46)	1.05 (0.86-1.29)	1.04 (0.85-1.27)
Ethnicity						
Non-Hispanic	6 047 932 (79.2)	1675 (86.6)	2.77	1 [Ref]	1 [Ref]	1 [Ref]
Hispanic	399 634 (5.2)	196 (10.1)	4.9	1.77 (1.53-2.05)	2.23 (1.92-2.60)	2.20 (1.89-2.56)
Declined, unknown, or missing	1 187 498 (15.6)	64 (3.3)	0.54	0.19 (0.15-0.25)	0.46 (0.34-0.62)	0.44 (0.33-0.60)
BMI						
<18.5	53 502 (0.7)	36 (1.9)	6.73	2.12 (1.51-2.98)	1.20 (0.86-1.69)	1.41 (1.00-1.98)
18.5 to <25	1 489 834 (19.5)	472 (24.4)	3.17	1 [Reference]	1 [Reference]	1 [Reference]
25 to <30	2 776 740 (36.4)	607 (31.4)	2.19	0.69 (0.61-0.78)	0.87 (0.77-0.98)	0.80 (0.71-0.91)
30 to <35, obesity I	2 060 412 (27.0)	459 (23.7)	2.23	0.70 (0.62-0.80)	1.02 (0.90-1.17)	0.93 (0.81-1.06)
35 to <40, obesity II	870 541 (11.4)	229 (11.8)	2.63	0.83 (0.71-0.97)	1.23 (1.04-1.45)	1.11 (0.94-1.31)
≥40, obesity III	384 035 (5.0)	132 (6.8)	3.44	1.08 (0.89-1.32)	1.44 (1.18-1.77)	1.31 (1.07-1.61)
Charlson Comorbidity Index score						
0	3 458 776 (45.3)	133 (6.9)	0.38	1 [Reference]	1 [Reference]	1 [Reference]
1	1 240 165 (16.2)	178 (9.2)	1.44	3.73 (2.98-4.67)	2.83 (2.24-3.58)	2.74 (2.18-3.45)
2	817 095 (10.7)	192 (9.9)	2.35	6.11 (4.90-7.62)	3.68 (2.90-4.67)	3.61 (2.88-4.53)
3	650 971 (8.5)	208 (10.7)	3.2	8.31 (6.69-10.33)	4.17 (3.28-5.30)	4.23 (3.37-5.31)
4	432 001 (5.7)	224 (11.6)	5.19	13.49 (10.89-16.72)	5.77 (4.53-7.35)	6.09 (4.85-7.64)
5-6	551 479 (7.2)	412 (21.3)	7.47	19.44 (15.99-23.64)	6.60 (5.23-8.34)	7.38 (5.94-9.17)
7-8	275 809 (3.6)	291 (15.0)	10.55	27.47 (22.37-33.72)	7.02 (5.46-9.03)	8.65 (6.83-10.96)
≥9	208 768 (2.7)	297 (15.3)	14.23	37.05 (30.19-45.46)	7.32 (5.64-9.50)	10.31 (8.09-13.15)
Diabetes						
No	6 040 109 (79.1)	947 (48.9)	1.57	1 [Reference]	1 [Reference]	1 [Reference]
Yes	1 594 955 (20.9)	988 (51.1)	6.19	3.95 (3.62-4.32)	1.27 (1.14-1.42)	1.25 (1.12-1.39)
Chronic kidney disease						
No	7 061 331 (92.5)	1322 (68.3)	1.87	1 [Reference]	1 [Reference]	1 [Reference]
Yes	573 733 (7.5)	613 (31.7)	10.68	5.71 (5.19-6.29)	1.24 (1.10-1.38)	1.34 (1.20-1.50)
Congestive heart failure						
No	7 397 046 (96.9)	1578 (81.6)	2.13	1 [Reference]	1 [Reference]	1 [Reference]
Yes	238 018 (3.1)	357 (18.4)	15	7.04 (6.28-7.90)	1.46 (1.28-1.66)	1.90 (1.68-2.16)
CAN score						
≤30	1 317 303 (17.3)	71 (3.7)	0.54	1 [Reference]	1 [Reference]	1 [Reference]
>30-55	1 383 519 (18.1)	213 (11.0)	1.54	2.86 (2.18-3.74)	1.08 (0.81-1.46)	NA
>55-75	1 178 832 (15.4)	300 (15.5)	2.54	4.72 (3.65-6.12)	1.18 (0.87-1.59)	NA
>75-90	891 513 (11.7)	544 (28.1)	6.1	11.33 (8.85-14.51)	1.88 (1.39-2.54)	NA
>90-95	59 419 (0.8)	76 (3.9)	12.79	23.76 (17.19-32.84)	2.94 (2.03-4.27)	NA
>95-98	176 613 (2.3)	293 (15.1)	16.59	30.83 (23.79-39.96)	3.26 (2.36-4.51)	NA
99	56 603 (0.7)	208 (10.7)	36.75	68.43 (52.26-89.59)	5.26 (3.72-7.44)	NA
Missing	2 571 262 (33.7)	230 (11.9)	0.89	1.66 (1.27-2.17)	1.59 (1.17-2.17)	NA

Abbreviations: BMI, body mass index (calculated as weight in kilograms divided by height in meters squared); CAN, Care Assessment Needs; NA, not applicable.

^a^ Model with CAN score was simultaneously adjusted for all 10 characteristics shown, categorized as shown.

^b^ Model without CAN score was simultaneously adjusted for 9 characteristics, ie, all the characteristics shown in the Table except CAN score.

^c^ The other group included Asian individuals and Pacific Islander or Native Hawaiian individuals.

#### Model Performance and Validation

We split the cohort into an early training period (May 21 to September 30, 2020) and a subsequent testing period (October 1 to November 2, 2020) to examine model stability over time and identify potentially optimistic estimates of performance. We used area under the receiver operating characteristic curve (AUROC) to assess discrimination. We calculated the sensitivity of each of the prioritization strategies described in the next section (ie, the proportion of SARS-CoV-2–related deaths that would be correctly identified and potentially prevented by an effective vaccine) at the time of vaccination of 5%, 10%, 20%, 30%, 40%, and 50% of the population. Based on these numbers, we estimated the proportional reduction in SARS-CoV-2–related deaths per day and total number of deaths that would be achieved when vaccination reached these levels, assuming that vaccination is 90% effective at preventing SARS-CoV-2–related death by preventing fatal infections or converting fatal into nonfatal infections. This assumption is reasonable for the Pfizer and Moderna vaccines, which have reported efficacies of approximately 95% against symptomatic COVID-19 infection.^2,3^ To assess model calibration, we calculated the ratio of expected to observed events, calibration-in the large (CITL), and calibration slope.

#### Comparison of Different Prioritization Strategies

We compared the performance characteristics of the following prioritization strategies: (1) our COVIDVax model-based allocation, in which individuals are vaccinated sequentially based on model scores starting with the top 5% model scores, followed by the scores greater than 5% to 10%, greater than 10% to 20%, greater than 20% to 30%, greater than 30% to 40%, greater than 40% to 50%, and greater than 50%; (2) an age-based allocation, in which individuals are vaccinated in age groups starting with those 90 years and older, those 85 to younger than 90 years, those 80 to younger than 85 years, those 75 to younger than 80 years, those 70 to younger than 75 years, those 65 to younger than 70 years, those 60 to younger than 65 years, those 50 to younger than 60 years, and those 18 to younger than 50 years; and (3) the CDC-ACIP phased allocation, in which individuals are vaccinated first in phase 1b (age ≥75 years) followed by phase 1c (age 65-74 years or 18-64 years with a high-risk medical condition as listed by CDC-ACIP), followed by everyone else (phase 2). The age-based allocation was compared because it does not require any special tools to implement, and age is the factor most strongly associated with SARS-CoV-2–related mortality.^9^ We also considered strategies based on CCI alone, the CAN score alone, or the VA COVID-19 (VACO) Index,^8^ a VA-based model that estimates mortality in persons who test positive for SARS-CoV-2.

Frontline and essential workers, who are included in CDC-ACIP phases 1b and 1c respectively, cannot be easily identified and would tend to reduce the association of the CDC-ACIP strategy with SARS-CoV-2–related mortality. We assumed that these individuals would be offered vaccination in parallel with the high-risk groups under all 3 strategies with similarly high priority, thus having no net effect on the comparisons among strategies.

#### Model Implementation

We evaluated whether the Palantir data integration platform,^19^ which the VA is currently leasing, could incorporate all necessary data streams and execute our model to produce risk scores for all VA enrollees in real-time. All VA data on the platform remain owned and governed by the VA.

## Results

### Baseline Characteristics of VA Enrollees

Of 7 635 064 included VA enrollees, the mean (SD) and median (interquartile range) age were 66.2 (13.8) years and 68 (56-75) years, respectively, with a substantial proportion aged 65 years or older (4 426 939 [58.0%]), 75 years or older (2 024 622 [26.5%]), or 85 years or older (755 283 [9.9%]). Most VA enrollees were men (7 051 912 [92.4%]) and White individuals (4 887 338 [64.0%]), with 1 116 435 (14.6%) Black individuals and 399 634 (5.2%) Hispanic individuals. Most cohort members (4 176 288 [54.6%]) had a CCI of 1 or greater.

### COVIDVax Model Development

During the 165-day follow-up period, there were 2422 SARS-CoV-2–related deaths among cohort members (mortality, 1.92 deaths per 1 million participants per day), including 1935 deaths in the training subset and 487 deaths in the testing subset. The model was developed in the training subset. A total of 6 candidate predictor variables (urban/rural location, cirrhosis, COPD, hypertension, MI, and PVD) were eliminated using the variable selection methods described previously. The remaining 10 candidate variables (ie, sex, age, race, ethnicity, BMI, CCI, diabetes, CKD, CHF, and CAN score) were statistically significantly associated with the outcome and included in the model (Table 1). Model coefficients appear in eTable 1 in the Supplement. Because the CAN score is not available outside the VA, we also developed a model that excluded the CAN score (Table 1).

### COVIDVax Model Performance

The model exhibited excellent discrimination with an AUROC of 85.3% (95% CI, 84.6%-86.1%) in the training and 83.6% (95% CI, 82.0%-85.3%) in the testing subset (Figure and Table 2). Dropping the CAN score reduced the AUROC only minimally (from 83.6% to 83.4% in the testing subset). The AUROC of a model that used only age was significantly lower (72.6%; 95% CI, 71.6%-73.6%), as was the AUROC for models that used only the CCI score, only the CAN score, or the VACO Index (eTable 2 in the Supplement). An AUROC could not be calculated for the CDC-ACIP strategy due to its broad categories. The model also performed well in subgroups defined by age, sex, race, ethnicity, and geographic region (Table 2).

**Figure.**
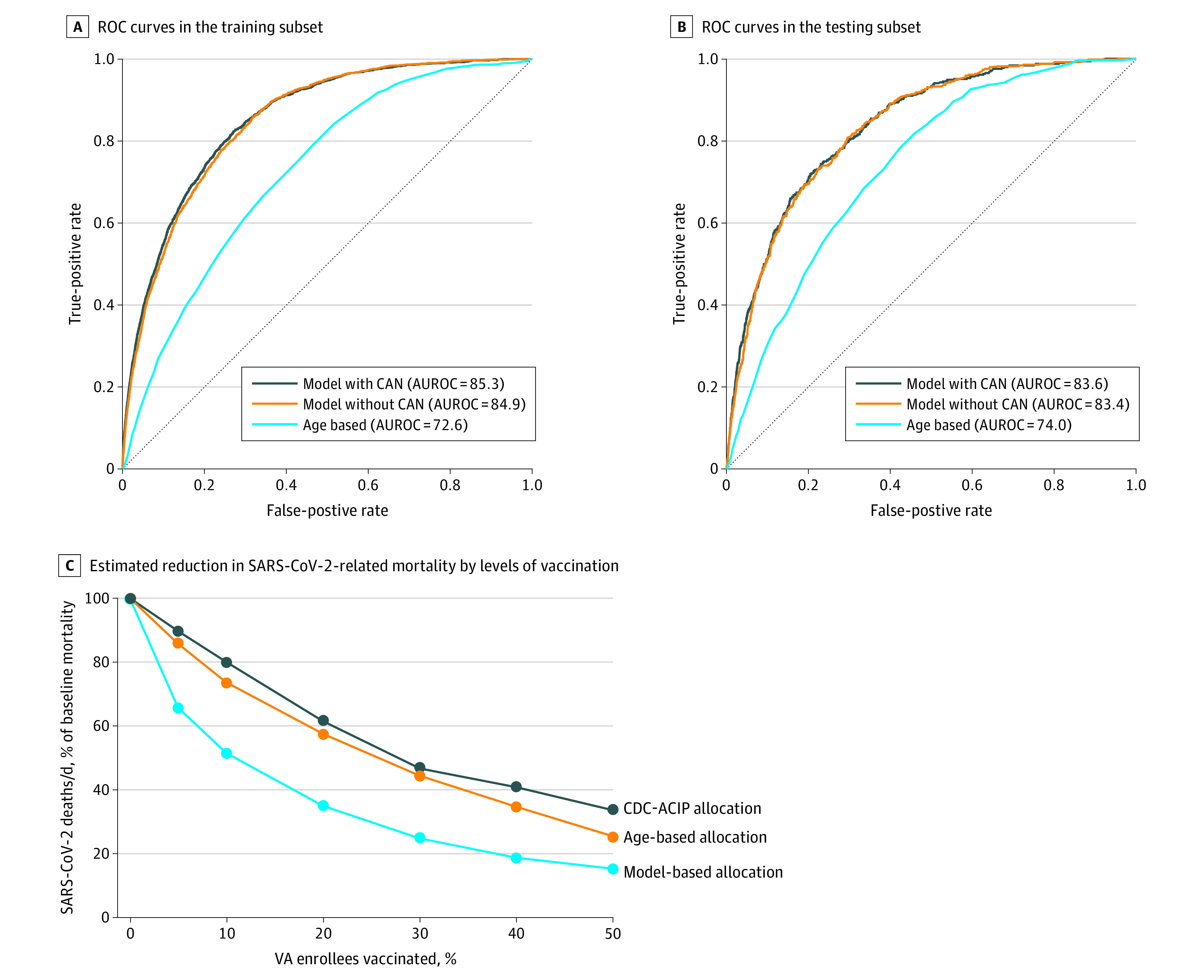
Receiver Operating Characteristic (ROC) Curve for Models Predicting SARS-CoV-2–Related Death Between May and October 2020 in Training and Testing Subsets and the Estimated Reduction in SARS-CoV-2–Related Mortality Achieved by Different Vaccination Prioritization Strategies C, The knots (ie, the points corresponding to 5%, 10%, 20%, 30%, 40%, and 50% vaccination levels) were plotted based on the sensitivity for detecting death at each vaccination level as shown in Table 3. For example, given that the model with CAN scores identifies 54% of deaths at the top 10% of scores, it means that when 10% of the population has been vaccinated, 54% of subsequent deaths would be prevented assuming that the vaccine is 100% effective. Assuming the vaccine is only 90% effective, then 0.54 × 0.90 (or 48.6%) of subsequent deaths would be prevented. Therefore, at that point, the mortality rate would be 1 − 0.486 (or 51.4%) of the baseline rate, which is the point plotted corresponding to 10% vaccination. An approximately constant rate of vaccinating was assumed, with the assumption that persons within each category would be vaccinated in no particular order, rather than in the strict order of the scores within each category. Therefore, a straight line joins the knots rather than a fitted curve. The area above each curve represents the proportion of deaths prevented by vaccination with each allocation strategy compared with no vaccination, as different levels of population vaccination are reached. The area between the curves is the proportion of deaths prevented by 1 allocation strategy vs another. The actual number of deaths prevented can be calculated by using the actual number of deaths per day at the beginning (before vaccination) in a given health care system or population and the time it would take to reach a certain level of population vaccination. For example, if a system/population such as the VA has 20 deaths per day and would take 150 days (5 months) to vaccinate 50% of the population, then 3000 deaths would occur by day 150 without any vaccination, of which 63.5% (n = 1905) would be prevented by vaccination using the COVIDVax model vs only 1233 (41.1%) by the CDC-ACIP phased allocation and 1368 (45.6%) by age-based allocation.

**Table 2.** AUROC for Models Predicting SARS-CoV-2–Related Death Among 7.6 Million Veterans Affairs Enrollees Compared With Age-Based Allocation^a^

Group	AUROC (95% CI)		
	Model		Age-based allocation^d^
	With CAN score^b^	Without CAN score^c^	
All persons			
Training subset	85.3 (84.6-86.1)	84.9 (84.1-85.6)	72.6 (71.6-73.6)
Testing subset	83.6 (82.0-85.3)	83.4 (81.8-85.0)	74.0 (72.1-75.9)
Age, y			
≥85	77.4 (75.8-79.0)	76.3 (74.6-77.9)	52.9 (50.9-55.0)
65 to <85	80.3 (79.2-81.3)	79.5 (78.5-80.5)	58.0 (56.6-59.5)
<65	83.5 (81.0-86.0)	83.5 (81.1-86.0)	67.2 (63.9-70.5)
Sex			
Women	88.3 (83.4-93.2)	87.6 (83.0-92.3)	77.9 (71.7-84.2)
Men	84.3 (83.6-85.1)	83.9 (83.2-84.6)	71.7 (70.8-72.6)
Race			
Black	84.5 (82.9-86.1)	84.3 (82.8-85.9)	77.1 (75.1-79.1)
American Indian or Alaska Native	75.4 (66.8-84.0)	75.6 (66.9-84.2)	65.8 (57.1-74.4)
Race other than Black, American Indian, or Alaska Native	85.0 (84.2-85.8)	84.5 (83.7-85.3)	73.3 (72.3-74.3)
Ethnicity			
Hispanic	81.3 (78.9-83.7)	80.9 (78.5-83.2)	74.8 (71.9-77.7)
Non-Hispanic	85.0 (84.3-85.8)	84.6 (83.9-85.3)	73.0 (72.1-73.9)
Geographic region^e^			
Northeast	85.0 (83.4-86.7)	84.7 (83.1-86.3)	74.8 (72.8-76.8)
Southeast	84.8 (83.7-85.9)	84.2 (83.1-85.3)	74.0 (72.6-75.4)
Midwest	85.7 (84.2-87.2)	85.4 (83.9-86.9)	72.8 (70.8-74.8)
West	85.9 (84.0-87.7)	85.4 (83.6-87.2)	71.8 (69.3-74.3)

Abbreviations: AUROC, area under the receiver operating characteristic curve; BMI, body mass index; CAN, Care Assessment Need; CCI, Charlson Comorbidity Index.

^a^ Training period extended from May 21, to September 30, 2020, and the testing period from October 1 to November 2, 2020.

^b^ Model with CAN score was simultaneously adjusted for all 10 characteristics shown in Table 1, ie, sex, age, race, ethnicity, BMI, CCI, diabetes, chronic kidney disease, congestive heart failure, and CAN score.

^c^ Model without CAN score was simultaneously adjusted for 9 characteristics, ie, all the characteristics shown in Table 1 except CAN score: sex, age, race, ethnicity, BMI, CCI, diabetes, chronic kidney disease.

^d^ Age-based allocation is the strategy of vaccinating the oldest first.

^e^ Northeast included Connecticut, DC, Delaware, Illinois, Indiana, Massachusetts, Maryland, Maine, Michigan, Missouri, New Hampshire, New Jersey, New York, Ohio, Pennsylvania, Rhode Island, Vermont, and Wisconsin; Southeast included Alabama, Arkansas, Florida, Georgia, Kentucky, Louisiana, Mississippi, North Carolina, Puerto Rico, South Carolina, Tennessee, Virginia, and West Virginia; Midwest included Colorado, Iowa, Kansas, Minnesota, Montana, North Dakota, Nebraska, Oklahoma, South Dakota, Texas, and Wyoming; and West included Alaska, Arizona, California, Hawaii, Idaho, New Mexico, Nevada, Oregon, Utah, and Washington.

Table 3 compares the sensitivity of COVIDVax-based with age-based or CDC-ACIP–based allocation strategies at different levels of vaccination. These analyses demonstrate that the model is more effective at identifying the small proportion of VA enrollees with the highest risk scores, among whom a disproportionately large number of SARS-CoV-2–related deaths occurred. For example, when 5% (approximately 382 000) or 10% ( approximately 763 000) of VA enrollees are vaccinated based on the highest model scores, then those who received vaccines would include 38.2% and 54.0% of the subsequent SARS-CoV-2–related deaths, respectively. In contrast, levels of vaccination of 5% or 10% based on age-only allocation (ie, oldest first) would include 16.0% and 29.4% of SARS-CoV-2–related deaths. We estimated that by the time 50% of the population is vaccinated, prioritizing vaccinees based on the COVIDVax model would result in 22.4% fewer SARS-CoV-2–related deaths (63.5% vs 41.1%) than an approach based on CDC-ACIP allocation phases and 17.9% fewer deaths (63.5% vs 45.6%) than an approach based on age alone (Table 3). eTable 3 in the Supplement shows that our model sensitivity was similar for identifying deaths that occurred during the entire 165-day follow-up period vs using only the first 55 or 110 days.

**Table 3.** Sensitivity of Each Prioritization Strategy at Different Vaccination Levels

Proportion of VA enrollees vaccinated, %	VA enrollees vaccinated, No.	Sensitivity of each vaccine prioritization strategy, %^a^				Deaths prevented by vaccination with each prioritization strategy, %^b^			
		Model		Age-based	CDC-ACIP phases	Model		Age-based	CDC-ACIP phases
		With CAN	Without CAN			With CAN	Without CAN		
5	381 579	38.2	35.3	16.0	11.8	17.2	15.9	7.2	5.3
10	763 556	54.0	51.7	29.4	22.7	29.3	27.5	13.8	10.4
20	1 526 995	72.3	71.1	47.4	42.9	43.1	41.4	24.2	20.0
30	2 291 586	83.4	82.5	61.9	59.4	52.1	50.6	32.5	28.7
40	3 054 788	90.4	90.5	72.7	65.9	58.6	57.4	39.5	35.6
50	3 816 650	94.2	94.5	82.9	73.9	63.5	62.6	45.6	41.1

Abbreviations: CAN, Care Assessment Need; CDC-ACIP, US Centers for Disease Control and Prevention Advisory Committee on Immunization Practices; SARS-CoV-2, severe acute respiratory syndrome coronavirus 2; VA, Veterans Affairs.

^a^ Sensitivity refers to the proportion of SARS-CoV-2–related deaths that occurred during the 165-day observation period that would be correctly identified by each strategy at different levels of vaccination of the population (ie, what proportion of deaths occurred in the first 5%, 10%, 20%, 30%, 40% and 50% of the population selected by each strategy).

^b^ The percentage of deaths prevented by vaccination (compared with no vaccination) is the area above each curve in the Figure, C as a proportion of the total area (ie, the total number of deaths that would occur without vaccination).

The Figure, C and Table 3 show how SARS-CoV-2–related mortality (deaths per day) would decline when different levels of vaccination are reached, calculated using the sensitivity values reported in Table 3. The area under each curve is the proportion of deaths that would occur, and the area above each curve is the proportion of deaths that would be prevented compared with no vaccination, in which case deaths per day are assumed to remain at baseline. The Figure, C shows that SARS-CoV-2–related mortality would decline much faster with the COVIDVax model’s prioritization, resulting in a much greater proportion of deaths prevented (Table 3).

Model calibration measures in the testing data were excellent and similar between the model with CAN, the model without CAN, and the age-based allocation strategies with ratios of expected to observed events of 93.0%, 93.7%, and 95.5%; CITL values of 0.072, 0.065, and 0.046; and calibration slopes of 0.928, 0.932, and 1.061, respectively.

### Model Implementation and Execution

The data platform was used to ingest all necessary VA data streams, execute the model, and generate a dashboard (eFigure in the Supplement) of individuals stratified by risk of SARS-CoV-2–related death for vaccination outreach. We also developed a web-based calculator that executes the COVIDVax model^20^ and provided all coefficients for others to execute it (eTable 1 in the Supplement).

## Discussion

The findings of this study suggest that a vaccination strategy that prioritizes individuals most likely to die would minimize SARS-CoV-2–related mortality during vaccine rollout. We developed a model that uses 10 baseline characteristics to estimate the risk of SARS-CoV-2–related death among more than 7.6 million VA enrollees. The model had excellent performance characteristics (AUROC, 85.3%) in a recent (May to November 2020) population-based cohort. We estimated that by the time 50% of the population is vaccinated, prioritizing vaccinees based on the model would result in 22.4% fewer SARS-CoV-2–related deaths than an approach based on CDC-ACIP allocation phases and 17.9% fewer deaths than an approach based on age alone. Our findings suggest that health care systems, such as VA, that have the capability to do so should consider implementing our model, and the CDC-ACIP should consider modification or substratification of their proposed allocation phases to better capture risk of SARS-CoV-2–related mortality.

The model demonstrated high sensitivity for SARS-CoV-2–related death even at low levels of vaccination, which would result in substantial reductions in SARS-CoV-2–related mortality even after small proportions of the population have been vaccinated (Figure, C). Table 3 and the Figure, C illustrate that we can estimate the proportion of deaths prevented only as a function of the proportion of persons vaccinated. The actual numbers of deaths prevented would be greater the longer it takes to vaccinate and the higher the absolute death rate without vaccination. For example, if it takes 150 days to vaccinate 50% of VA enrollees, in whom approximately 20 deaths per day were occurring at baseline, then 3000 (150 × 20) deaths would occur without vaccination, 1905 deaths (63.5%) would be prevented by COVIDVax-based vaccination, but only 1233 (41.1%) would be prevented by CDC-ACIP phased allocation. Alternatively, if it takes 200 days to vaccinate 50% of VA enrollees and the baseline mortality is higher at approximately 30 deaths per day (given the surge in SARS-CoV-2–related mortality since the study’s observation period), then 6000 (200 × 30) deaths would occur without vaccination, 3810 (63.5%) would be prevented by model-based vaccination and 2460 (41.1%) by CDC-ACIP phased allocation. Given that more than 2500 SARS-CoV-2–related deaths per day have been reported in the United States since December 15, 2020.^21^ and that vaccination rollout has been slower than expected,^22^ a very large number of deaths can be prevented by strategies that directly model and prioritize high-risk persons.

Many models perform well in silico but fail to be implemented because the predictor variables are not readily available or the modeling algorithms are too complicated (eg, neural network models) for real-world execution. We demonstrated that the data integration platform that the VA is currently using can ingest all necessary data streams and execute the model in real time for all current VA enrollees. We envision that this platform may be used to identify and continually update high-risk persons for vaccination outreach, to track vaccinated persons and those remaining unvaccinated, to match supply and demand for the vaccine across VA networks and facilities, and to track real-world vaccine effectiveness on a single platform. We developed a model that did not include the CAN score for use in settings other than the VA and a web-based calculator^20^ to estimate risk in individuals for vaccine prioritization.

Prioritization based on our model would adhere to the 4 ethical principles outlined by ACIP. It maximizes benefits (by targeting those at highest risk for vaccination), promotes justice (by identifying older adults or those with a high comorbidity burden who will require focused outreach for vaccination), mitigates health inequities (by assigning higher priority to racial and ethnic minorities directly reflecting their higher risk of mortality), and promotes transparency (by using an evidence-based model with explicit parameters). CDC-ACIP phases also include prioritization of frontline workers (phase 1b) and essential workers (phase 1c), which is unrelated to risk of SARS-CoV-2–related death but justified because of the societal impacts of these groups. These groups can still be prioritized in parallel with a model-based, risk-based prioritization strategy.

### Limitations

This study has limitations. Our calculations underestimate the overall vaccination benefit because they do not account for the beneficial consequences on those unvaccinated through lowered transmission. It is hard to measure and model an individual’s risk of transmitting SARS-CoV-2, but we assume that the impacts of the different risk-based allocation strategies on transmission are broadly similar given that none are aimed specifically at reducing transmission. Our model is population based because it was derived from all 7.6 million persons enrolled in VA care. However, VA enrollees are older and more likely to be male, to have comorbid conditions, and to have adverse social determinants of health. In contrast, they have access to comprehensive, high-quality health care. To determine the extent to which our model results are generalizable to other populations, the model will need to be externally validated.

## Conclusions

In this study, we developed and internally validated a model predicting SARS-CoV-2–related mortality among all VA enrollees that can be used to prioritize persons for vaccination. The model would potentially result in substantial reductions in mortality compared with the allocation strategies currently proposed by the CDC-ACIP.

## Notes

Supplement. **eMethods. **Imputation of Missing BMI Values **eTable 1. **Coefficients of the Logistic Regression Models (With and Without CAN Score) Used to Estimate the Risk of SARS-CoV-2–Related Death During the Period From May 21 to September 30, 2020, Among All VA Enrollees Who Were at Risk as of May 21, 2020 **eTable 2. **Comparison of the Area Under the Receiver Operating Characteristic Curve (AUROC) of the Model to the AUROC of Using Age-Only, Charlson Comorbidity Index–Only, CAN Score–Only, or VACO Index for Predicting SARS-CoV-2–Related Death **eTable 3. **Sensitivity of Model-Based Allocation at Different Observation Periods at the Time of Vaccination of 5%, 10%, 20%, 30%, 40% and 50% of the Population **eFigure. **Screenshots of Data Integration Platform Executing the Model and Identifying High-Risk Persons for Vaccination Prioritization
