# Supplementary material for: Development of COVIDVax Model to Estimate the Risk of SARS-CoV-2–Related Death Among 7.6 Million US Veterans for Use in Vaccination Prioritization
Source: JAMA Netw Open. 2021 Apr 6;4(4):e214347. doi: 10.1001/jamanetworkopen.2021.4347 (PMC8025111; doi:10.1001/jamanetworkopen.2021.4347)
Supplement: Supplement. — eMethods. Imputation of Missing BMI Values eTable 1. Coefficients of the Logistic Regression Models (With and Without CAN Score) Used to Estimate the Risk of SARS-CoV-2–Related Death During the Period From May 21 to September 30, 2020, Among All VA Enrollees Who Were at Risk as of May 21, 2020 eTable 2. Comparison of the Area Under the Receiver Operating Characteristic Curve (AUROC) of the Model to the AUROC of Using Age-Only, Charlson Comorbidity Index–Only, CAN Score–Only, or VACO Index for Predicting SARS-CoV-2–Related Death eTable 3. Sensitivity of Model-Based Allocation at Different Observation Periods at the Time of Vaccination of 5%, 10%, 20%, 30%, 40% and 50% of the Population eFigure. Screenshots of Data Integration Platform Executing the Model and Identifying High-Risk Persons for Vaccination Prioritization [file jamanetwopen-e214347-s001.pdf]

Supplemental Online Content

Ioannou GN, Green P, Fan VS, et al. Development of COVIDVax model to estimate the risk of SARS-CoV-2–related death among 7.6 million US veterans for use in vaccination prioritization. *JAMA Netw Open*. 2021;4(4):e214347. doi:10.1001/jamanetworkopen.2021.4347

eMethods. Imputation of Missing BMI Values

eTable 1. Coefficients of the Logistic Regression Models (With and Without CAN Score) Used to Estimate the Risk of SARS-CoV-2–Related Death During the Period From May 21 to September 30, 2020, Among All VA Enrollees Who Were at Risk as of May 21, 2020

eTable 2. Comparison of the Area Under the Receiver Operating Characteristic Curve (AUROC) of the Model to the AUROC of Using Age-Only, Charlson Comorbidity Index–Only, CAN Score–Only, or VACO Index for Predicting SARS-CoV-2–Related Death

eTable 3. Sensitivity of Model-Based Allocation at Different Observation Periods at the Time of Vaccination of 5%, 10%, 20%, 30%, 40% and 50% of the Population

eFigure. Screenshots of Data Integration Platform Executing the Model and Identifying High-Risk Persons for Vaccination Prioritization

This supplemental material has been provided by the authors to give readers additional information about their work.

© 2021 Ioannou GN et al. *JAMA Network Open.*

eMethods: Imputation of Missing BMI Values

Imputation of missing BMI values.

Missing BMI values were deterministically imputed. Data for BMI were assumed to be missing at random after independently extracting BMI and setting any BMI measured after the index date to missing among those tested. This assumption was verified using observed data. Based on the large sample size of non-missing BMI, a truncated normal distribution was chosen using the 1st and 99th percentiles as left and right truncation values respectively. Missing BMI values were imputed using a truncated normal model with sex, age, race, ethnicity, urban/rural, Charlson Comorbidity Index, diabetes, CKD, and CHF in addition to SARS-CoV-2 related death. The CAN score was omitted from the imputation to allow more generalizability in use outside the VA system. All missing BMI values were successfully imputed.

© 2021 Ioannou GN et al. *JAMA Network Open.*

eTable 1. Coefficients of the Logistic Regression Models (With and Without CAN Score) Used to Estimate the Risk of SARS-CoV-2–Related Death During the Period From May 21 to September 30, 2020, Among All VA Enrollees Who Were at Risk as of May 21, 2020

	Adjusted Odds Ratio: COVIDVax Model with CAN†	Adjusted Odds Ratio: COVIDVax Model without CAN‡
**Intercept**	-12.014492	-11.889342
**Sex**		
Female (%)	0.00000000	0.00000000
Male (%)	0.51083778	0.59931844
**Age (years)**		
18 to <50	0.00000000	0.00000000
50 to <60	0.40134529	0.38878105
60 to <65	0.85950678	0.86474941
65 to <70	1.09484487	1.17620040
70 to <75	1.36252803	1.43285275
75 to <80	1.47945709	1.77775682
80 to <85	1.66217641	2.01115747
85 to <90	1.74963691	2.37366036
≥90	2.19762683	2.82412673
**Race**		
White	0.00000000	0.00000000
Black	0.67976632	0.65816496
American Indian/Alaska Native	0.87568408	0.88150793
Other	0.26799874	0.24404052
Declined/Unknown /Missing	0.05227075	0.03695410
**Ethnicity**		
Non-Hispanic	0.00000000	0.00000000
Hispanic	0.80426634	0.78963438
Declined/Unknown /Missing	-0.77293989	-0.81167892
**BMI (kg/m^2)**		
<18.5	0.17363500	0.33002839
18.5 to <25	0.00000000	0.00000000
25 to <30	-0.17149557	-0.24998726
30 to <35 (Obese I)	0.01571945	-0.08188009
35 to <40 (Obese II)	0.18947128	0.09100135
≥40 (Obese III)	0.39166030	0.29784097
**Charlson Comorbidity Index**		
0	0.00000000	0.00000000

© 2021 Ioannou GN et al. *JAMA Network Open.*

1	1.03878070	1.00796154
2	1.30194976	1.28258629
3	1.42620967	1.44202350
4	1.75068854	1.80520631
5-6	1.88523578	1.99696773
7-8	1.94666167	2.15599625
≥9	1.98926119	2.33143941
**Diabetes**		
No	0.00000000	0.00000000
Yes	0.24028275	0.21990963
**Chronic Kidney Disease**		
No	0.00000000	0.00000000
Yes	0.21189320	0.29614684
**Congestive heart failure**		
No	0.00000000	0.00000000
Yes	0.37668438	0.64074342
**Care Assessment Need (CAN) Score***		
≤ 30	0.00000000	-
> 30 to 55	0.08156707	-
> 55 to 75	0.16290607	-
> 75 to 90	0.62849712	-
> 90 to 95	1.07734732	-
> 95 to 98	1.17952757	-
≥ 99	1.65729469	-
Missing	0.46448187	-

† Model with CAN: Logistic regression model that simultaneously adjusted for 10 characteristics as shown in the Table: sex, age, race, ethnicity, body mass index (BMI), Charlson Comorbidity Index, diabetes, chronic kidney disease, congestive heart failure and the Care Assessment Need (CAN) score
‡ Model without CAN: Logistic regression model that simultaneously adjusted for 9 characteristics shown in the Table: sex, age, race, ethnicity, body mass index (BMI), Charlson Comorbidity Index (CCI), diabetes, chronic kidney disease and omitted the CAN score

© 2021 Ioannou GN et al. *JAMA Network Open.*

eTable 2. Comparison of the Area Under the Receiver Operating Characteristic Curve (AUROC) of the Model to the AUROC of Using Age-Only, Charlson Comorbidity Index–Only, CAN Score–Only, or VACO Index for Predicting SARS-CoV-2–Related Death

	AUROC (95% CI)					
	**COVIDVax Model with CAN†**	**COVIDVax Model without CAN‡**	**Age-Based§ Allocation**	**Charlson Comorbidity Index††**	**CAN Score****	**VACO Index‡**
Training subset*	85.3 (84.6- 86.1)	84.9 (84.1- 85.6)	72.6 (71.6- 73.6)	79.7 (78.8-80.6)	77.4 (76.2-78.6)	78.7 (77.8-79.5)
Testing subset*	83.6 (82.0- 85.3)	83.4 (81.8- 85.0)	74.0 (72.1- 75.9)	78.7 (76.7-80.7)	75.2 (72.7-77.7)	79.6 (77.9-81.2)

* Training period extended from 05/21/20 to 9/30/20 and the testing period from 10/1/20 to 11/02/20.
§ Age-based allocation is the strategy of vaccinating the oldest first, categorized as ≥90, 85 to <90, 80 to <85, 75 to <80, 70 to <75, 65 to <70, 60 to <65, 50 to <60, and 18 to <50.
† COVIDVAX Model with CAN: Model that simultaneously adjusted for all 10 characteristics: sex, age, race, ethnicity, body mass index (BMI), Charlson Comorbidity Index (CCI), diabetes, chronic kidney disease, congestive heart failure and the Care Assessment Need (CAN) score
‡ COVIDVAX Model without CAN: Model that simultaneously adjusted for 9 characteristics: sex, age, race, ethnicity, body mass index (BMI), Charlson Comorbidity Index (CCI), diabetes and chronic kidney disease and omitted the CAN score.
††The Charlson comorbidity index is categorized as 0,1,2,3,4,5-6, 7-8, ≥9
**CAN score is the Care Assessment Needs score, which is a validated measure of 1-year mortality in VA enrollees that is calculated using socio-demographics, clinical diagnoses, vital signs, medications, laboratory values, and health care utilization data from VA's national EHR. The CAN score was recently shown to be a predictor of COVID-19-related mortality[1].
‡‡ The VACO Index used sex, age, Charlson comorbidity Index and history of MI or PVD and was designed to predict mortality in persons who test positive for SARS-CoV-2 in the VA healthcare system[2]

© 2021 Ioannou GN et al. *JAMA Network Open.*

eTable 3. Sensitivity of Model-Based Allocation at Different Observation Periods at the Time of Vaccination of 5%, 10%, 20%, 30%, 40% and 50% of the Population

		Sensitivity* of the COVIDVax model-based prioritization over different lengths of observation period		
**Proportion of VA Enrollees Vaccinated**	**Number of VA Enrollees Vaccinated**	**55 Days**	**110 Days**	**165 Days**
5%	381,579	43.1	39.9	38.2
10%	763,556	59.3	56.3	54.0
20%	1,526,995	74.8	73.8	72.3
30%	2,291,586	85.0	84.9	83.4
40%	3,054,788	92.0	91.4	90.4
50%	3,816,650	95.1	94.8	94.2

* Sensitivity refers to the proportion of SARS-CoV-2-related deaths that occurred during the observation period that would be correctly identified at different percentage levels of vaccination of the population (i.e. what proportion of deaths occurred in the “top” 5%, 10%, 20%, 30%, 40% and 50% of the population selected by each strategy)

© 2021 Ioannou GN et al. *JAMA Network Open.*

eFigure. Screenshots of Data Integration Platform Executing the Model and Identifying High-Risk Persons for Vaccination Prioritization

Dashboard view of our model execution showing examples of redacted lists of high-risk persons (defined as top 10% of model-estimated risk), together with the estimated risk score for each person, that could be used for identifying and contacting persons for vaccination at each VISN and facility (results shown for VISN 20)

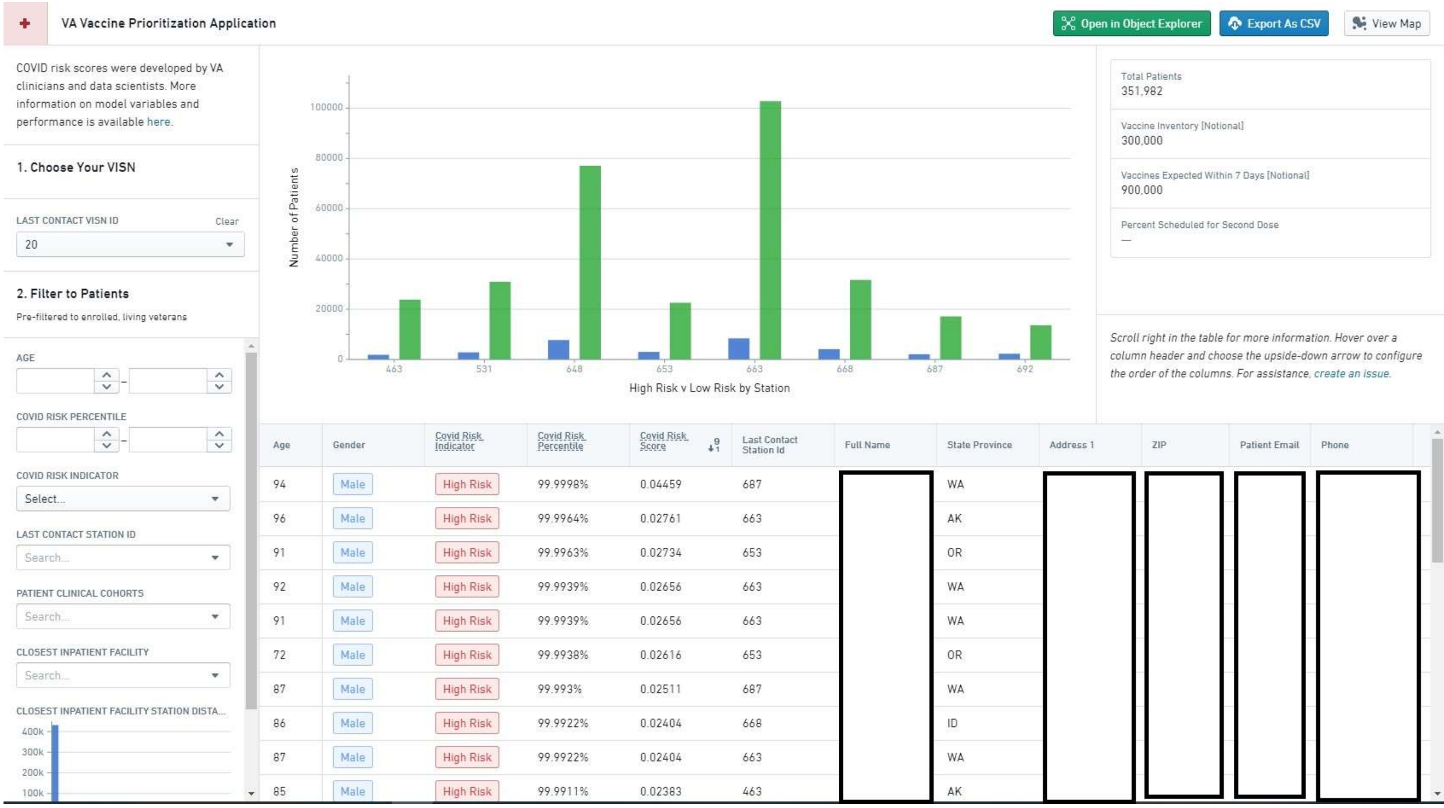

1. Osborne TF, Veigulis ZP, Arreola DM, Roosli E, Curtin CM. Automated EHR score to predict COVID-19 outcomes at US Department of Veterans Affairs. *PLoS One.* 2020;15(7):e0236554.
2. King JT, Jr., Yoon JS, Rentsch CT, et al. Development and validation of a 30-day mortality index based on pre-existing medical administrative data from 13,323 COVID-19 patients: The Veterans Health Administration COVID-19 (VACO) Index. *PLoS One.* 2020;15(11):e0241825.

© 2021 Ioannou GN et al. *JAMA Network Open.*
